# Five-year real-world outcomes of anti-vascular endothelial growth factor monotherapy versus combination therapy for polypoidal choroidal vasculopathy in a Chinese population: a retrospective study

**DOI:** 10.1186/s12886-019-1245-4

**Published:** 2019-11-21

**Authors:** Jingyuan Yang, Mingzhen Yuan, Erqian Wang, Song Xia, Youxin Chen

**Affiliations:** 1 Department of Ophthalmology, Peking Union Medical College Hospital, Peking Union Medical College, Chinese Academy of Medical Sciences, No.1 Shuaifuyuan, Wangfujing, Dongcheng District, Beijing, 100730 China; 2 Key Laboratory of Ocular Fundus Diseases, Chinese Academy of Medical Sciences, Beijing, China; 3 Department of Ophthalmology, Guizhou Provincial People’s Hospital, Guiyang, China

**Keywords:** Anti-vascular endothelial growth factor therapy, Combination therapy, Photodynamic therapy, Polypoidal choroidal vasculopathy, Retinal pigment epithelium, Visual acuity

## Abstract

**Background:**

To evaluate 5-year outcomes of anti-vascular endothelial growth factor (VEGF) monotherapy and combination therapy of anti-VEGF agents and photodynamic therapy (PDT) for polypoidal choroidal vasculopathy (PCV) in a real-world Chinese population.

**Methods:**

Retrospective study. Fifty-three eyes of 46 patients with subtype 1 and 2 PCV followed up for at least 60 months were grouped into three regimens: anti-VEGF monotherapy, PDT combining with anti-VEGF therapy initially, and PDT combining with deferred anti-VEGF therapy. Main outcome measure was best-corrected visual acuity (BCVA) using logarithm of minimal angle of resolution (logMAR).

**Results:**

The mean BCVA of eyes with subtype 1 PCV (*n* = 28) deteriorated from 0.69 logMAR at baseline to 1.25 logMAR at months 60 (*P* = 0.001), while the mean BCVA of eyes with subtype 2 PCV (*n* = 25) sustained stable from 0.62 logMAR at baseline to 0.57 at months 60 (*P* = 0.654). No significant differences of visual outcomes were found between the 3 treatment regimens for subtype 1 PCV. Anti-VEGF monotherapy and initial combination treatment had better visual outcomes in eyes with subtype 2 PCV than deferred combination group during part of follow-up significantly. Initial combination group needed a less number of PDT than deferred combination group (*P* < 0.001).

**Conclusions:**

Compared with subtype 1 PCV, subtype 2 PCV has a more favorable visual outcome in real world. All the regimens presented unfavorable visual outcomes for subtype 1 PCV. Anti-VEGF monotherapy and initial combination therapy should be superior to deferred combination therapy in the long-term management of subtype 2 PCV. Prospective randomized studies of larger size are needed to determine the long-term efficacy and safety of various treatment for PCV in real world.

## Background

Polypoidal choroidal vasculopathy (PCV) has been recognized as a major cause of significant and acquired vision loss in elderly patients [1–3]. PCV is characterized by polypoidal or aneurysmal hyperfluorescence on indocyanine green angiography (ICGA) [4, 5]. Branching vascular network (BVN) and serosanguinous pigment epithelium detachment are frequently associated with polypoidal lesions [6, 7]. PCV is a subtype of neovascular age-related macular degeneration (nvAMD), and PCV also falls in the pachychoroid spectrum diseases [6].

Although its pathogenesis is still unknown, anti-vascular endothelial growth factor (VEGF) and photodynamic therapy (PDT) have been used for treating PCV in real world. Unlike typical nvAMD, where intravitreal anti-VEGF therapy has been the mainstay of treatment for over a decade, currently anti-VEGF therapy, verteporfin PDT, and various combinations of these therapies for PCV exist simultaneously [8–12]. In order to achieve the best possible visual outcomes, both EVEREST-II and PLANET studies indicated that anti-VEGF therapy and combination therapy with PDT give excellent functional visual outcomes at 1 year [9, 12]. Although long-term favorable visual outcome with PDT and anti-VEGF therapy has been validated respectively, [13–19] little is known about that, among anti-VEGF monotherapy and various combination of PDT and anti-VEGF therapy, which treatment is superior with respect to long-term best-corrected visual acuity (BCVA) outcome in real world. Regression of polyps is a significant aspect of treatment, nevertheless identifying the treatment that is superior for visual outcome was regarded more important to patients than polyp regression [6, 7, 10].

In this study, we evaluated long-term visual outcomes at least 5 years after anti-VEGF monotherapy and combination of PDT and anti-VEGF therapy for various subtypes of PCV in real world. We also investigated imaging morphological features associated with long-term visual outcomes in these patients.

## Methods

### Enrollment of study subjects

We retrospectively reviewed 53 eyes of 46 consecutive patients with treatment-naïve PCV who fulfilled the inclusion and exclusion criteria. The patients were treated at the Department of Ophthalmology of Peking Union Medical College Hospital between January 2012 and February 2014. All patients completed at least 5 years of follow-up after the first treatment. This retrospective study was approved by the Institutional Review Board of Peking Union Medical College Hospital and was conducted in accordance with the tenets of the Declaration of Helsinki. Patient characteristics were retrieved from their medical charts, including age at initial diagnosis, gender. Inclusion criteria were: (1) presence of polypoidal lesions with or without BVN on ICGA; (2) symptomatic PCV with leakage on fluorescein angiography (FA); and (3) follow-up at least 60 months after initial treatment. Exclusion criteria were: (1) any other treatment for PCV including laser photocoagulation, transpupillary thermotherapy, and radiotherapy; (2) other concomitant ocular diseases, such as vein or artery occlusion, diabetic retinopathy, pathologic myopia, and glaucoma; and (3) any systemic contraindications to angiographic dyes. PCV diagnosis was based on ICGA findings of the polypoidal lesions with or without BVN within 6 min after dye injection.

### Examinations

We collected the examination data from the baseline visit and the 1-, 2-, 3-, 6-, 12-, 18-, 24-, 30-, 36-, 42-, 48-, 54-, and 60-month follow-ups and interpreted them retrospectively. Main outcome measurement was BCVA. Eyes gaining or losing logarithm of minimal angle of resolution (logMAR) of more than 0.3 at 60 months were classified into the improved or deteriorated group, respectively, while the others were classified into the stable group. BCVA was converted to logMAR for statistical analysis from decimal visual acuity using a decimal visual acuity chart. FA/ICGA (Spectralis HRA, Heidelberg Engineering, Heidelberg, Germany) was performed at baseline. BCVA measurement and optical coherence tomography (OCT) examination (Spectralis OCT, Heidelberg Engineering, Heidelberg, Germany; 3D OCT-2000 or Triton, Topcon, Tokyo, Japan) were performed at every visit. Greatest linear dimension (GLD) was determined by the ICGA, which included entire polyps and BVNs at the early phase of ICGA, assessed using HRA built-in software. PCV was classified into 2 subtypes according to appearances on ICGA and OCT: polypoidal CNV as subtype 1 PCV, and typical PCV as subtype 2 PCV [20]. Formation of polypoidal lesions was classified into 2 categories: isolated and interconnected (cluster or string). Number of polypoidal lesions was also classified into 2 categories: single and multiple. OCT features at baseline and months 60 included the presence of intraretinal fluid and subretinal fluid, and the continuity of external limiting membrane (ELM), ellipsoid zone (EZ) and retinal pigment epithelium (RPE).

### Intervention

Patients underwent various interventions were enrolled and grouped into 3 groups. Anti-VEGF therapy was recommended to every patient. Patients in the anti-VEGF monotherapy group underwent injections of anti-VEGF drugs, including Ranibizumab, Bevacizumab, and Conbercept. Standard PDT was considered in eyes in which there was leakage from both polyps and BVN, or extensive subretinal fluid or exudation associated with PED was noticed [7]. In the PDT in combination with initial anti-VEGF therapy group (initial combination group) and PDT in combination with deferred anti-VEGF therapy group (deferred combination group), the initial combination regimen consisted of a session of PDT guided by ICGA and an anti-VEGF injection within 7 days after PDT or on the same day, while the deferred combination regimen consisted of anti-VEGF injections after more than 7 days from initial PDT. After the initial treatment, repeat treatment of anti-VEGF therapy was applied as needed (pro re nata [PRN]). We applied retreatment when BCVA decreased exceeding one line using decimal visual chart, or retinal hemorrhage, retinal edema, and subretinal fluid were observed without treatment. Conversive therapy was considered to be performed from one anti-VEGF agent to another anti-VEGF agent when no significant response and resistance were noticed. No significant response and resistance were considered as lack of improvement, mainly including angiographic leakage and exudative changes on OCT despite further repetitive injections of one anti-VEGF agent. The decision was at the physicians’ discretion in our department. Each treatment was explained detailedly to the patients until patients and us reached an agreement on the treatment plan.

### Statistical analysis

SPSS 25.0 (IBM, Chicago, Illinois, USA) was used for statistical analysis. A paired and 2-sample *t*-test and one-way analysis of variance (ANOVA) with LSD method were used for analysis of continuous variables. Chi-square test, Kruskal-Wallis test, and Wilcoxon signed-rank test were used for categorical variables. Pearson and Spearman correlation analysis were used for investigating correlation. Multiple linear regression analysis was performed on related imaging features at baseline (subtype of PCV, continuity of ELM, EZ, and RPE, intraretinal fluid, subretinal fluid, GLD, distance from the center of foveola to the nearest polyp and BVN, and the number and formation of polyps), and the BCVA at months 60 and the changes of BCVA were used as the dependent variable using the stepwise model with the threshold *P* value = 0.05 for enter and 0.10 for remove, in which age, gender, and BCVA at baseline were adjusted. Differences with *P* < 0.05 were considered statistically significant.

## Results

In total, 53 eyes of 46 patients who had completed at least 60 months of follow-up visits after initial treatment were enrolled. Patients had a mean age of 63.30 ± 10.10 years and showed a male predominance (34 male, 64.2%). Baseline clinical details are listed in Table 1. Twenty-eight eyes received anti-VEGF monotherapy, and 25 eyes received combination therapy, in which 14 eyes received initial combination therapy, and 11 eyes received deferred combination therapy. There were no significant differences of baseline clinical characteristics among three treatment regimens except that the anti-VEGF monotherapy group had greater proportion of presence of subretinal fluid than the initial combination group did significantly (21/28 vs 4/10, *P* = 0.012, Chi-square test) (Additional file 1: Table S1).

**Table 1:** Baseline characteristics of the patients with polypoidal choroidal vasculopathy

Baseline characteristics	
Age of initial diagnosis (year), mean (SD)	63.30 (10.10)
Female/male, n	34/19
Subtype 1/ subtype 2, n	28/25
Best-corrected visual acuity (logMAR), mean unit (SD)	0.67 (0.48)
Greatest linear dimension (μm), mean (SD)	3277 (2510)
Formation of polyps	
Isolated, n (%)	27 (50.94%)
Interconnected, n (%)	26 (49.06%)
Number of polyps	
Single, n (%)	14 (26.42%)
Multiple, n (%)	39 (73.58%)
Distance from foveola to the nearest polyp (μm), mean (SD)	1342 (1208)
Distance from foveola to branching vascular network (μm), mean (SD)	256 (455)
Continuous external limiting membrane, n (%)	6 (11.3)
Continuous ellipsoid zone, n (%)	4 (7.5)
Continuous retinal pigment epithelium, n (%)	23 (43.4)
Intraretinal fluid, n (%)	30 (56.6)
Subretinal fluid, n (%)	30 (56.6)

*logMAR* logarithm of minimal angle of resolution, *SD* standard deviation

### Visual outcomes

For all eyes enrolled, the mean BCVA was 0.67 ± 0.48 logMAR at baseline and 0.95 ± 0.72 logMAR at months 60. The mean BCVA during follow-up remained not inferior to baseline BCVA until months 54 (all *P*-values > 0.05, paired *t*-test), and the mean BCVA at months 54 and 60 after initial treatment was worse than the baseline BCVA (*P* = 0.021 and 0.007, respectively, paired *t*-test).

**Fig. 1:**
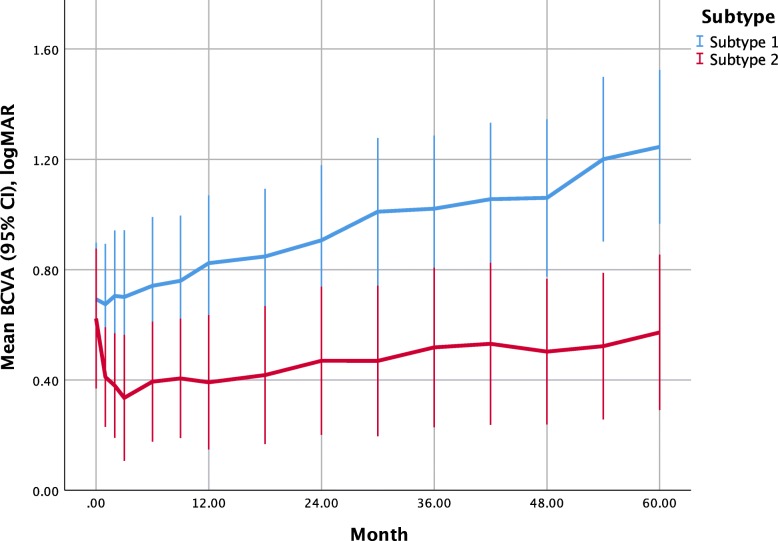
Mean best-corrected visual acuity (BCVA) (95% confidence interval, CI) using logarithm of the minimal angle of resolution (logMAR) of subtype 1 and 2 polypoidal choroidal vasculopathy (PCV). The mean BCVA of subtype 1 PCV deteriorated significantly during the follow-up period, while the mean BCVA of subtype 2 PCV sustained stable

The BCVA of eyes with subtype 1 PCV at baseline and at months 60 were 0.69 ± 0.49 and 1.25 ± 0.66 logMAR, respectively, which deteriorated significantly (*P* = 0.001, 2-sample *t*-test) (Fig. 1). The BCVA of eyes with subtype 2 PCV at baseline and at months 60 were 0.62 ± 0.55 and 0.57 ± 0.62, which sustained stable (*P* = 0.783, 2-sample *t*-test). The eyes with subtype 1 and 2 PCV had similar mean BCVA at baseline (*P* = 0.654, 2-sample *t*-test), but the eyes with subtype 2 PCV had significantly better BCVA at months 60 than the eyes with subtype 1 PCV (*P* = 0.001, 2-sample *t*-test). Among the eyes with subtype 1 PCV, no significant differences of the values of BCVA were found between various treatment regimens at baseline and the follow-up period (all *P*-value > 0.05). Among the eyes with subtype 2 PCV, the values of BCVA in the anti-VEGF monotherapy were better than that in the deferred combination group significantly at months 1, 2, 3, 6, and 30 (*P* = 0.024, 0.007, 0.014, 0.011, and 0.024, respectively, ANOVA with LSD method), and the value of BCVA in the initial combination group at months 30 was better than that in the deferred combination group significantly (*P* = 0.047, ANOVA with LSD), while no significant differences of the values of BCVA were found between various treatment regimens at baseline and the other follow-up period (all *P*-value > 0.05).

**Fig. 2:**
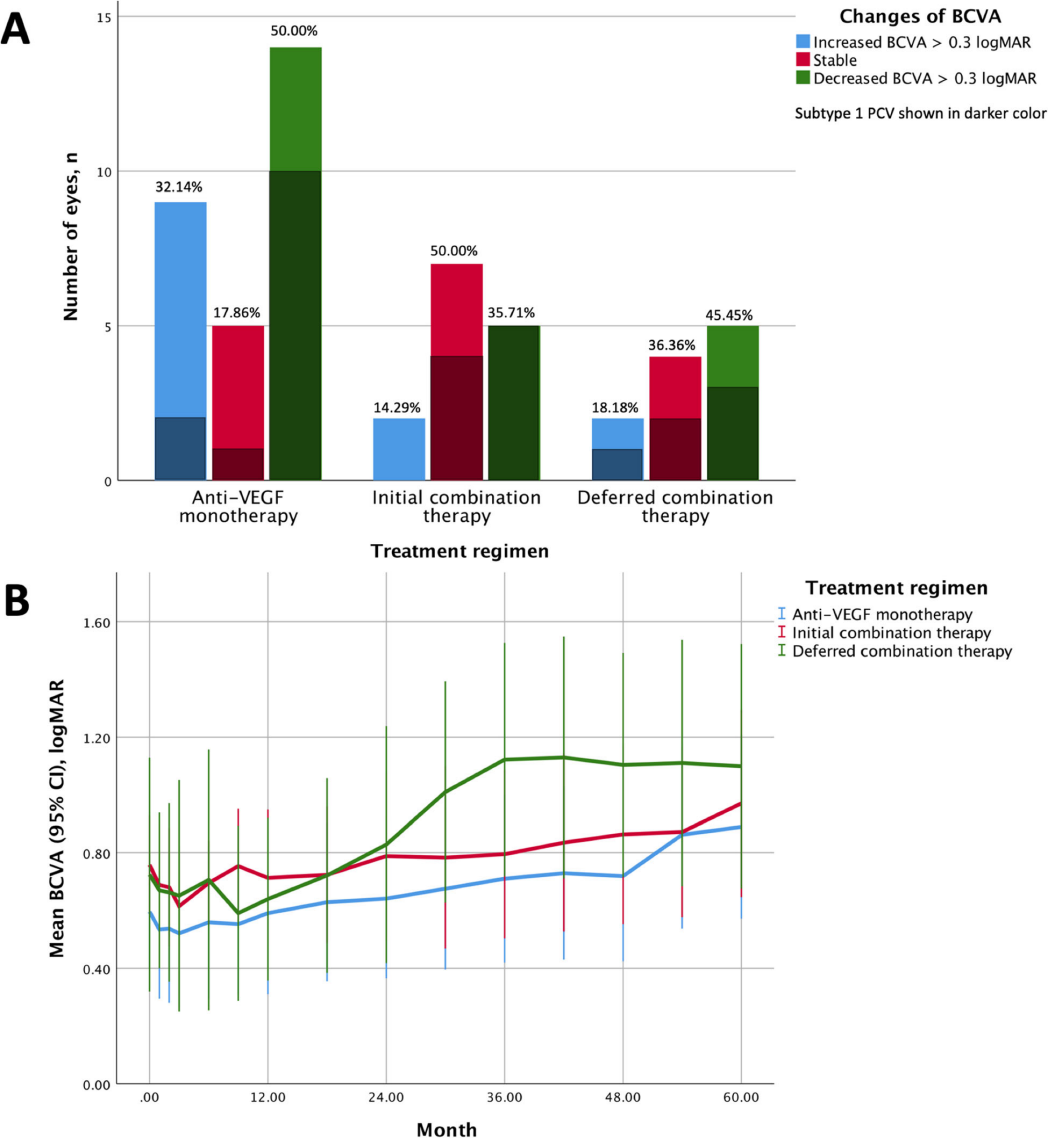
Changes of best-corrected visual acuity (BCVA) (95% confidence interval, CI) during the flollow-up period using various treatment regimens for both subtype 1 and 2 polypoidal choroidal vasculopathy. **a** The anti-vascular endothelial growth factor (VEGF) monotherapy had the greatest proportion of improved BCVA, while the initial combination therapy had the greatest proportion of stable BCVA. The anti-VEGF monotherapy had similar proportion of deteriorated BCVA to the deferred combination therapy. **b** No significant differences of BCVA were noticed at baseline and month 60 among various treatment regimens

The proportions of various groups of visual outcomes were presented in Fig. 2. No significant differences of the proportion of improved, stable, and deteriorated BCVA were found between various treatment regimens in eyes with either subtype 1 or 2 PCV (all *P*-value > 0.05, Kruskal-Wallis test).

### Treatment during follow-up

Table 2 shows the mean number of anti-VEGF therapy and PDT in various treatment regimens during the follow-up period. The number of eyes with subtype 1 and 2 in anti-VEGF monotherapy group, initial combination group, and deferred combination group was 13 and 15, 9 and 5, and 6 and 5, respectively. The percentage of conversion of anti-VEGF drugs was 41.51%. No significant differences of BCVA at baseline and months 60, BCVA changes, and the number of PDT were noticed between eyes with and without conversive therapy (*P* = 0.068, 0.060, 0.539, and 0.086, 2-sample *t*-test), and the eyes with conversive treatment needed less number of anti-VEGF injections than the eyes without conversive treatment significantly (*P* < 0.001). No significant difference of the total number of anti-VEGF therapy was detected between various groups in either eyes with subtype 1 or eyes with subtype 2 PCV (all *P*-values > 0.05, ANOVA with LSD method). A significantly greater number of PDT was needed in the deferred combination group than that in the initial combination group (*P* = 0.025, 2-sample *t*-test).

**Table 2:** Mean (SD) number of anti-VEGF therapy and PDT annually over the course of 60 months

	Anti-VEGF monotherapy group		Initial combination group		Deferred combination group	
	Anti-VEGF	PDT	Anti-VEGF	PDT	Anti-VEGF	PDT
The first year	2.36 (1.95)	0 (0)	3.00 (2.80)	1.00 (0)	1.09 (1.38)	1.09 (0.30)
The second year	0.75 (1.14)	0 (0)	1.21 (1.67)	0 (0)	1.27 (1.35)	0.36 (0.50)
The third year	0.79 (1.50)	0 (0)	0.93 (1.38)	0 (0)	0.64 (1.12)	0.09 (0.30)
The fourth year	0.50 (1.14)	0 (0)	1.14 (1.96)	0.14 (0.36)	1.00 (1.26)	0.36 (0.67)
The fifth year	0.25 (0.70)	0 (0)	0.79 (0.80)	0 (0)	1.18 (1.33)	0 (0)
Total	4.64 (4.08)	0 (0)	7.07 (4.97)	1.14 (0.36)	5.18 (3.60)	1.91 (0.94)

*PDT* photodynamic therapy, *SD* standard deviation, *VEGF* vascular endothelial growth factor

The mean total number of anti-VEGF therapy on eyes with subtype 1 PCV was greater than that on eyes with subtype 2 PCV with borderline significance (6.75 vs 4.23, *P* = 0.058, 2-sample *t*-test). No significant difference of the total number of PDT was found between subtype 1 and 2 PCV (0.75 vs 0.43, *P* = 0.244, 2-sample *t*-test).

### Imaging morphology features

The number of eyes with isolated polyps and interconnected polyps was 17 and 11 in the anti-VEGF monotherapy group, 7 and 7 in the initial combination group, and 3 and 8 in the deferred combination group. The number of eyes with single polyps and multiple polyps was 7 and 21 in the anti-VEGF monotherapy group, 4 and 10 in the initial combination group, and 3 and 8 in the deferred combination group. At the final visit, the presence of intraretinal fluid, subretinal fluid, and macular atrophy on OCT images were noticed in 20, 11, and 14 eyes, respectively.

Multiple linear regression analysis revealed that the BCVA at months 60 was associated with the formation of polyps and the continuity of RPE (β = 0.671, *P* < 0.001, and β = − 0.455, *P* = 0.007, respectively) (Additional file 2: Figure S1), and the changes of BCVA was also associated with the formation of polyps and the continuity of RPE (β = 0.659, *P* < 0.001, and β = − 0.459, *P* = 0.007, respectively). The eyes with deteriorated BCVA had less percentage of continuous RPE at baseline than the eyes with improved and stable BCVA (*P* = 0.003 and 0.046, respectively, Wilcoxon signed-rank test). Among the eyes with continuous RPE, the values of mean BCVA in the anti-VEGF monotherapy group were better than that in the deferred combination group significantly at months 12, 30, 36, 42, and 48 (*P* = 0.032, 0.007, 0.010, 0.024, and 0.041, respectively, ANOVA with LSD method), and the value of mean BCVA in the initial combination group at months 30 was better than that in the deferred combination group significantly (*P* = 0.010, ANOVA with LSD method).

## Discussion

In this retrospective study, we compared the five-year outcomes of anti-VEGF monotherapy and combination therapy for various subtypes of PCV. Fifty-three treatment-naïve PCV eyes treated with anti-VEGF monotherapy and combination therapy were analyzed. The subtype 2 PCV had a favorable visual outcome, while the subtype 1 PCV had a relatively unfavorable visual outcome. Anti-VEGF monotherapy and initial combination therapy achieved more favorable visual outcome compared with deferred combination therapy, especially for subtype 2 PCV. Initial combination therapy needed less number of PDT than deferred combination therapy. Imaging morphological features, including the continuity of RPE at baseline, were associated with the decision of treatment and the visual outcomes at months 60, which could be regarded as imaging biomarkers that might predict response to therapy.

We compared anti-VEGF monotherapy and combination therapy, which were recommended by recent guideline and studies [6, 7, 9, 12]. In 2 recent randomized controlled trial (RCT), EVERST-II and PLANET studies which used different anti-VEGF agents, both therapy regimens with 12-month duration were compared [9, 12]. EVERST-II study demonstrated that combination therapy had superior visual and angiographic outcomes, and a less number of anti-VEGF therapies [9]. However, no comparison on long-term outcomes of anti-VEGF therapy and combination therapy was performed. Additionally, the patients in EVEREST-II study was assigned into various groups randomly, which is scarcely possible in real world because differences in individual characteristics of PCV eyes and financial burdens should be considered when deciding on the best treatment option for each patient. Besides, compared with EVEREST-II study, PLANET study concludes that no additional benefit in combining with PDT as a rescue therapy, which suggested that the outcome of initial combination regimen of anti-VEGF therapy and PDT might differ from that of deferred combination regimen of anti-VEGF agents plus deferred PDT [12]. Similarly, the combination therapy in the present study was divided into 2 groups as well: the initial combination and deferred combination. When comparing these treatment regimens, no significant differences of baseline clinical features were detected except subretinal fluid, which was not considered in decision on various treatment regimens and has not been proved, as a structural feature, to be related to long-term visual acuity outcomes directly.

Several studies have reported various long-term visual outcomes of PCV treated with various regimens. Saito et al. retrospectively evaluated the 60-month outcome of PDT with or without anti-VEGF therapy for PCV in 60 eyes [18]. The mean logMAR visual acuity at baseline was 0.66 and after 60 months was 0.71. Kang et al. reported an improved visual outcome from a mean baseline BCVA of 0.78 logMAR to the 5-year BCVA of 0.67 logMAR in 42 PCV eyes treated with PDT [17]. On the contrary, unfavorable visual acuity was also detected in another study reported Chang et al. They retrospectively evaluated the 4-year treatment outcome of 31 PCV eyes treated with anti-VEGF therapy [15]. The mean visual acuity at baseline was 0.52 logMAR and after 4 years was 0.83 logMAR. However, all the previous studies did not classify enrolled eyes into various subtypes. In the present study, we classified the enrolled eyes into 2 subtypes according to criteria provided by previous studies [20–22]. The eyes of subtype 1, polypoidal CNV, had an unfavorable visual outcome, which deteriorated from 0.69 to 1.25 logMAR significantly, while the eyes of subtype 2, typical PCV, had a favorable visual outcome, which improve from 0.62 to 0.57 logMAR without significant difference. Jang et al. also reported a better BCVA at baseline and at months 12 after the initial treatment in eyes with subtype 2 PCV than that in eyes with subtype 1 PCV [20]. The present study revealed a long-term course of treatment on PCV, and suggested that various subtypes of PCV in real world presented totally different long-term visual outcomes.

The mean number of anti-VEGF therapies in the anti-VEGF monotherapy group was 4.64, which was less than that of previous studies. An extensive study of LAPTOP study for totally 5 years reported a mean injection number of 14.8 for ranibizumab in the anti-VEGF monotherapy group, in which the number of ranibizumab injection was 8.0 in the last 3 years after the LAPTOP study, while the number of aflibercept was 3.7 [23]. The injection number of conversive therapy was less than the injection number of only ranibizumab. Another Japanese study reported a mean injection number of 18.2 for ranibizumab [19]. The mean injection number of anti-VEGF monotherapy in the present study was much less than the previous studies. Conversive therapy might help reduce the injection number. Besides, eyes with less severity and activity was treated with anti-VEGF monotherapy rather than combination therapy in real world according to guidelines, [7] which might lead to a less number of injections. Additionally, in order to reduce the selection bias due to the limitations of retrospective studies, not only eyes needed persistent treatment were enrolled, eyes with favorable visual outcomes were also enrolled, which might contribute the less injection number. Furthermore, the enrolled Chinese patients might bear the financial burden, and the anti-VEGF agents used in this study have not been paid by medical insurance during majority of the follow-up period, which indeed reduced the patients’ therapeutic compliance. What’s more, the retreatment criteria in the present study was not totally same as that of previous studies, [18, 19, 23] and the treatment decision were at the discretion of the treating physicians rather than a strict prospective protocol. And some patients with lesions of less activity and severity were allowed to visit us every 6 months. For majority eyes enrolled in the present study, only explicit signs of recurrence rather than potential signs were present, retreatment would be performed on the eyes showing response to treatment. Therefore, comparing with previous studies, patients might receive anti-VEGF therapies of less injection numbers in real world, especially in developing countries.

The present study revealed differences on the changes of BCVA at various time points and treatment number between initial and deferred combination regimen, which suggested that they should be analyzed respectively. PDT has been proved to be effective in the management of PCV [7]. And in real world, initial PDT with deferred anti-VEGF therapy has been performed. However, its long-term outcomes have not been investigated. In the present study, the initial combination group requires a significantly less number of PDT than the deferred combination group. When considering the similar BCVA at baseline, the initial combination regimen had a more favorable BCVA outcome at months 30 in eyes with continuous RPE and in eyes of subtype 2 PCV. And the initial combination regimen was more likely to earn improved and stable BCVA outcomes than deferred combination regimen. Therefore, when combination regimen was needed, initial combination of PDT and anti-VEGF therapy would be recommended by the present study.

Imaging morphological features associated with outcomes were also investigated. Firstly, we divided eyes into 2 subtypes, and we found that the subtype 2 PCV had a much more favorable visual acuity. Secondly, deteriorated BCVA has more percentage of discontinuous RPE. Neurosensory retina might be affected by abnormal vessels or BVN directly when RPE was discontinuous, and the higher percentage of discontinuous RPE might contribute to the deteriorated BCVA outcomes. At the meantime, we found that, the eyes with continuous RPE treated with anti-VEGF monotherapy had a better visual outcome than the eyes treated with deferred combination therapy during the follow-up period. PDT could lead to choriocapillary occlusion, RPE and neuroretina injury [24, 25]. Therefore, we speculated that the dysfunction of RPE and outer layers neuroretina after PDT without anti-VEGF therapy might lead to an unfavorable visual outcome. On the contrary, anti-VEGF agents can alleviate the inflammation reaction, [26, 27] which might protect retina and choriocapillary in the eyes of initial combination group. Thirdly, the formation of polyps was associated with BCVA outcomes. Polyps are a clinical characteristic of PCV, and regression of polyps plays an important role in the management of PCV [7, 10]. Suzuki et al. reported that multiple polyps showed more correlation with poorer BCVA, which suggested that the number of polyps might be valuable to understand the prognosis of PCV [28]. In the presented study, we noticed that the formation of polyps were also associated with visual outcomes. Hou et al. and Cackett et al. summarized 3 formation of polyps, including single, cluster, and string, but the relationship between the formation of polyps and visual outcomes was not investigated [29, 30]. Single polyps referred to isolated polyps, while cluster and string polyps referred to interconnected polyps. Therefore, we summarized the formation of polyps into 2 types, including the isolated and interconnected polyps. In the present study, interconnected polyps occurred more frequently in eyes of deteriorated BCVA. Polyps at baseline may reflect varying activity and severity of PCV, and further study will be needed to definitively determine the prognostic and mechanistic impact of polyps in PCV.

Our study has several limitations, including the relatively small patient number, especially in the initial and deferred combination therapy groups. Given the small numbers in these groups, it is difficult to draw firm conclusions in some situations. Because of the retrospective nature, it was difficult to obtain regular follow-up angiograms which did not affect decisions regarding retreatment. Another possible limitation is selection bias. In order to minimize potential selection, we enrolled eyes treated with different regimens and eyes with various visual outcomes. However, only Chinese patients were enrolled, and worldwide multicenter investigations might be needed to study PCV in real world. Additionally, although no RCTs demonstrated that any anti-VEGF agents were superior to the others, it is unknown the impact of conversion of anti-VEGF agents on the outcomes of PCV in the present study, we could not evaluate the different effects of various anti-VEGF agents on the visual outcomes.

## Conclusions

In conclusion, the 5-year outcomes of anti-VEGF monotherapy and combination therapy for PCV were reviewed. Our study demonstrated that anti-VEGF monotherapy and initial combination therapy were favorable overall over a 60-month follow-up period. Eyes with subtype 2 PCV and eyes with continuous RPE or isolated polyps were more likely to gain a favorable visual outcome. Decisions on treatment regimens should take the imaging features of PCV lesions into consideration. Because this study was a retrospective review with limited size, large, long-term, and prospective randomized studies are needed to determine the efficacy and safety profiles for patients with PCV of various severity and activity.

## Supplementary information

**Additional file 1: Table S1.** *P*-values of comparisons of baseline clinical characteristics in eyes with polypoidal choroidal vasculopathy performed three different regimens, including anti-VEGF monotherapy, initial combination therapy, and deferred combination therapy.

**Additional file 2: Figure S1.** Mean best-corrected visual acuity (BCVA) (95% confidence interval, CI) using logarithm of the minimal angle of resolution of polypoidal choroidal vasculopathy (PCV) which were classified according to the formation of polyps (A) and continuity of retinal pigment epithelium (B). No significant differences of BCVA were noticed at baseline regardless of the formation of polyps and continuity of retinal pigment epithelium. However, the eyes with isolated polyps or continuous retinal pigment epithelium had better BCVA at months 60 significantly.

## Data Availability

The datasets used and/or analyzed during the current study are available from the corresponding author on reasonable request.

## Abbreviations

ANOVA: analysis of variance
BCVA: best-corrected visual acuity
BVN: branching vascular network
ELM: external limiting membrane
EZ: ellipsoid zone
FA: fluorescein angiography
GLD: greatest linear dimension
ICGA: indocyanine green angiography
logMAR: logarithm of minimal angle of resolution
nvAMD: neovascular age-related macular degeneration
OCT: optical coherence tomography
PCV: polypoidal choroidal vasculopathy
PDT: photodynamic therapy
PRN: pro re nata
RCT: randomized controlled trial
RPE: retinal pigment epithelium
VEGF: vascular endothelial growth factor
