# Supplementary material for: Five-year real-world outcomes of anti-vascular endothelial growth factor monotherapy versus combination therapy for polypoidal choroidal vasculopathy in a Chinese population: a retrospective study
Source: BMC Ophthalmol. 2019 Nov 21;19:237. doi: 10.1186/s12886-019-1245-4 (PMC6873695; doi:10.1186/s12886-019-1245-4)
Supplement: Supplementary file 1 — Additional file 1: Table S1. P-values of comparisons of baseline clinical characteristics in eyes with polypoidal choroidal vasculopathy performed three different regimens, including anti-VEGF monotherapy, initial combination therapy, and deferred combination therapy. [file 12886_2019_1245_MOESM1_ESM.docx]

Additional file 1: **Table S1.** *P*-values of comparisons of baseline clinical characteristics in eyes with polypoidal choroidal vasculopathy performed three different regimens, including anti-VEGF monotherapy, initial combination therapy, and deferred combination therapy

|  | *P*-value |
| --- | --- |
| Best-corrected visual acuity (logMAR) | 0.536 |
| Greatest linear dimension (µm) | 0.387 |
| Distance from foveola to the nearest polyp (µm) | 0.056 |
| Distance from foveola to branching vascular network (µm) | 0.795 |
| Classification of formation of polyps | 0.339 |
| Classification of number of polps | 0.861 |
| Continuity of external limiting membrane | 0.478 |
| Continuity of ellipsoid zone | 0.720 |
| Continuity of retinal pigment epithelium | 0.687 |
| Presence of intraretinal fluid | 0.327 |
| Presence of subretinal fluid | 0.016 |
