# Supplementary figures and images for: Five-year real-world outcomes of anti-vascular endothelial growth factor monotherapy versus combination therapy for polypoidal choroidal vasculopathy in a Chinese population: a retrospective study
Source: BMC Ophthalmol. 2019 Nov 21;19:237. doi: 10.1186/s12886-019-1245-4 (PMC6873695; doi:10.1186/s12886-019-1245-4)

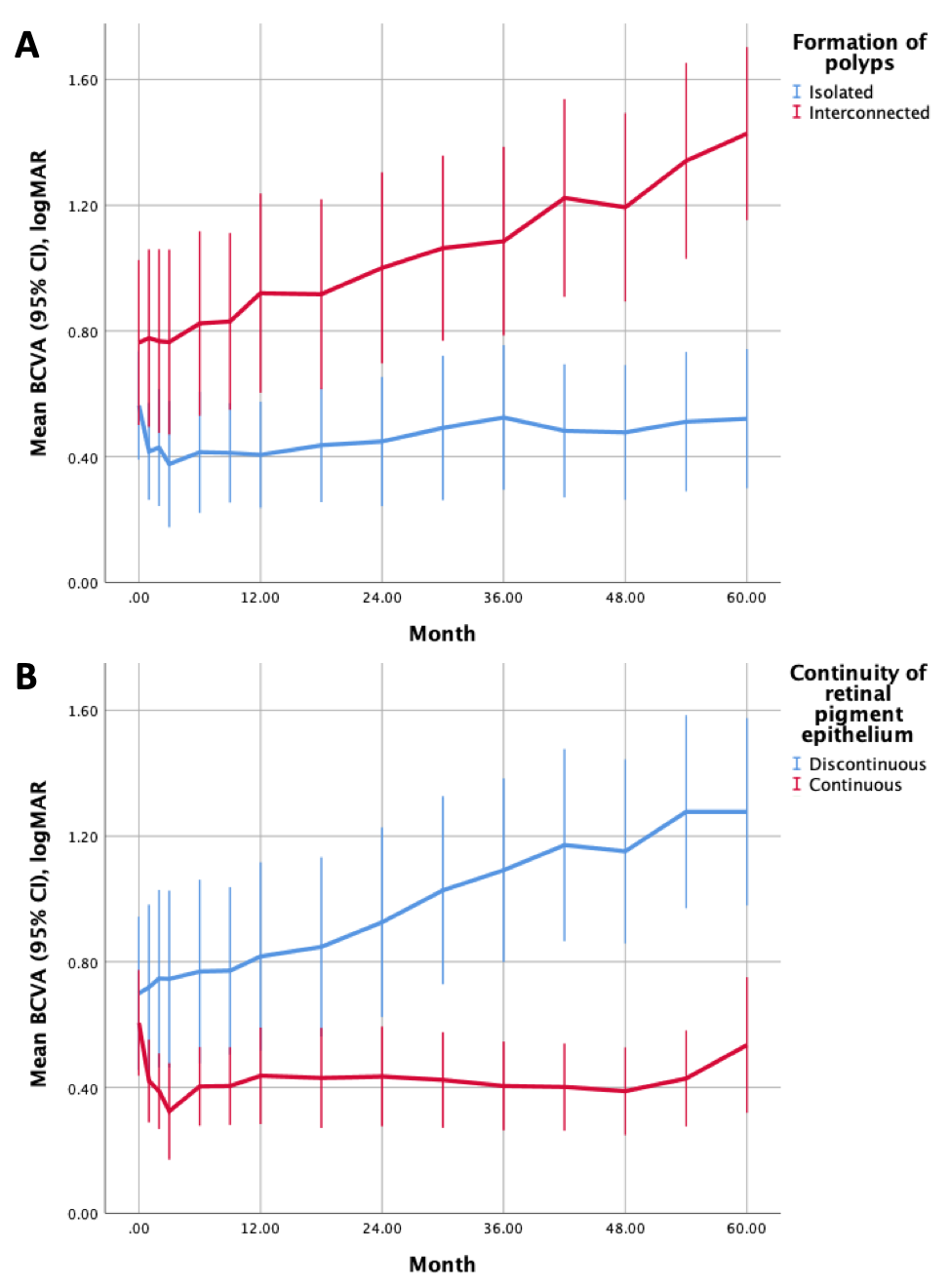

Supplement: Supplementary file 2 — Additional file 2: Figure S1. Mean best-corrected visual acuity (BCVA) (95% confidence interval, CI) using logarithm of the minimal angle of resolution of polypoidal choroidal vasculopathy (PCV) which were classified according to the formation of polyps (A) and continuity of retinal pigment epithelium (B). No significant differences of BCVA were noticed at baseline regardless of the formation of polyps and continuity of retinal pigment epithelium. However, the eyes with isolated polyps or continuous retinal pigment epithelium had better BCVA at months 60 significantly. [file 12886_2019_1245_MOESM2_ESM.tif]
